# Evaluation of a Neonatal Resuscitation Curriculum in Liberia

**DOI:** 10.3390/children6040056

**Published:** 2019-04-08

**Authors:** Mary P. Chang, Camila B. Walters, Carmelle Tsai, Deborah Aksamit, Francis Kateh, John Sampson

**Affiliations:** 1 Department of Emergency Medicine, University of Texas at Southwestern Medical Center, Dallas, TX 75390, USA; 2 Department of Anesthesiology/Pediatric Anesthesiology, Vanderbilt University Medical Center, Nashville, TN 37232, USA; camila.walters@vumc.org; 3 Department of Pediatrics, Division of Emergency Medicine, The Children’s Hospital of Philadelphia and University of Pennsylvania, Philadelphia, PA 19104, USA; tsaic@email.chop.edu; 4 Office of Emergency Training, Response, and Evaluation, Johns Hopkins School of Medicine, Baltimore, MD 21205; USA; daksami1@jhmi.edu; 5 Ministry of Health and Social Welfare, P. O. Box 10-9009 1000, Monrovia 10, Liberia; frankateh@aol.com; 6 Department of Anesthesiology/Critical Care Medicine, Johns Hopkins School of Medicine, Baltimore, MD 21205, USA; jsampso4@jhmi.edu

**Keywords:** neonatal resuscitation, newborn resuscitation, neonatal mortality, simulation training, cardiopulmonary resuscitation

## Abstract

Neonatal mortality in Africa is among the highest in the world. In Liberia, providers face significant challenges due to lack of resources, and providers in referral centers need to be prepared to appropriately provide neonatal resuscitation. A team of American Heart Association health care providers taught a two-day neonatal resuscitation curriculum designed for low-resource settings at a regional hospital in Liberia. The goal of this study was to evaluate if the curriculum improved knowledge and comfort in participation. The curriculum included simulations and was based on the Neonatal Resuscitation Protocol (NRP). Students learned newborn airway management, quality chest compression skills, and resuscitation interventions through lectures and manikin-based simulation sessions. Seventy-five participants were trained. There was a 63% increase in knowledge scores post training (*p* < 0.00001). Prior cardiopulmonary resuscitation (CPR) training, age, occupation, and pre-intervention test score did not have a significant effect on post-intervention knowledge test scores. The median provider comfort score improved from a 4 to 5 (*p* < 0.00001). Factors such as age, sex, prior NRP education, occupation, and post-intervention test scores did not have a significant effect on the post-intervention comfort level score. A modified NRP and manikin simulation-based curriculum may be an effective way of teaching health care providers in resource-limited settings. Training of providers in limited-resource settings could potentially help decrease neonatal mortality in Liberia. Modification of protocols is sometimes necessary and an important part of providing context-specific training. The results of this study have no direct relation to decreasing neonatal mortality until proven. A general resuscitation curriculum with modified NRP training may be effective, and further work should focus on the effect of such interventions on neonatal mortality rates in the region.

## 1. Introduction

In the last decade, substantial progress has been made in the reduction of under-five child mortality worldwide. The global under-five mortality rate was 93 deaths per 1000 live births in 1990 and decreased to 21 deaths per 1000 live births in 2017. Despite this, neonatal mortality rates remain a significant contributor to under-five mortality, actually increasing from 41% of all under-five deaths in 2000 to 46% in 2017 [1]. Africa continues to have a disproportionately high neonatal mortality rate of 26.7 per 1000 live births, compared to the global rate of 18.0 per 1000 live births in 2017 [2].

Liberia has faced significant challenges in improving health indicators, owing in part to its political and social history. The civil war from 1989 to 2003 left Liberia with a devastated infrastructure. Health systems and infrastructure were rebuilt from the ground up. Post-war Liberia suffered the devastation of the Ebola epidemic in 2014, resulting in yet another hit to the health care system [3]. Specifically with regards to neonatal health, the proportion of women delivering babies with a skilled health care provider declined from 52% in 2013 to 37% in 2014 [4]. Despite the aforementioned hardships, Liberia saw a significant decrease in the neonatal mortality rate from 34.2 per 1000 live births in 2006 to 23 per 1000 live births in 2016 [5]. As Liberia looks towards achieving the sustainable development goal (SDG) target to reduce neonatal mortality as low as 12 per 1000 live births by 2030, more neonatal resuscitation training program implementation will be necessary [6].

Since the majority of neonatal deaths are perinatal, and an estimated 10% of newborns require resuscitation at birth, addressing the neonatal mortality rate requires newborn resuscitation training. Standardized programs have been developed, most commonly the Neonatal Resuscitation Program (NRP), and specifically for the low-resource setting, Helping Babies Breathe (HBB). Multiple studies have been done looking at the efficacy of these programs, and meta-analyses reveal standardized programs are associated with decreased early neonatal mortality [7]. Preliminary data also suggest that simulation-based training improves teamwork behavior [8]. While there is a paucity of data on the baseline skillset of Liberian health care workers in neonatal resuscitation, data from other similarly resource-limited countries in Africa report a low level of neonatal resuscitation skills [9]. A systematic review and Delphi estimation of neonatal resuscitation in resource-limited settings, however, does suggest that facility-based (as opposed to community-based) neonatal resuscitation could reduce term intra-partum-related deaths by 30% [10]. Similarly, another study investigating the cost and impact of neonatal interventions in Asia and Africa estimates that skilled care at birth may avert 13–24% of neonatal deaths in resource-limited countries, and bundling skilled intrapartum care with additional emergency post-natal care for newborns could result in additional cost savings and mortality benefits [11]. Therefore, making additional progress towards decreasing the neonatal mortality rate in Liberia in a cost-effective and sustainable manner will necessarily involve not only formalized neonatal resuscitation intervention, but focused efforts on hospital facility-based programs in order to maximize neonate emergency care capacity. The goal of this study was to teach a modified neonatal resuscitation curriculum to staff at a Liberian hospital in a rural setting and evaluate the effectiveness of the teaching with knowledge and comfort with resuscitation skills surveys.

## 2. Materials and Methods

### 2.1. Setting

The Jackson F. Doe Memorial Regional Referral Hospital (JFD) is a regional hospital in Tappita, Liberia. It serves as a tertiary referral center, receiving patients from the entire country of Liberia as well as bordering countries of Cote d’Ivoire and Guinea. Nine hundred and twenty pediatric patients were admitted during the year this study was conducted. There is a four-bed intensive care unit where neonates can receive care. There are three incubators, and oxygen is available most of the time. There are no neonatal intensive care units in the country. One to two nurses staff the entire 30-bed ward with the help of one pediatrician, who is always on call.

Deliveries most often occur in the community, and newborns and neonates in distress may be brought to JFD for further management. A limited number of deliveries, including high-risk deliveries, occur at JFD. There are limited surgical staff available for obstetric emergencies, such as caesarean sections.

### 2.2. Design

A two-day general resuscitation curriculum for the staff at JFD was developed based on standard Basic Life Support (BLS), Advanced Cardiopulmonary Life Support (ACLS), Pediatric Advanced Life Support (PALS), and NRP protocols [12]. Adult resuscitation was taught on day one, and pediatric resuscitation, including for neonates, was taught on day two. To address the need at JFD for widely applicable training in a setting where both time and personnel are significantly limited, one integrated curriculum that would be suitable for nurses, residents, physicians, pharmacists, and support staff alike was developed. Accounting for JFD staff availability, the course directors opted to integrate an abbreviated NRP curriculum into the program, highlighting key skills most applicable to the JFD setting. Topics such as giving Narcan were not taught since it is not available in this setting, but most of the foundational topics were kept. The HBB curriculum was considered but not applied, as JFD is a hospital setting with the ability to support the usage of slightly more resource-intensive techniques like positive pressure ventilation, intravenous medication administration, and intubation.

The intervention utilized lectures, low-fidelity simulations with infant manikins, and hands-on medical equipment training. Simulator manikins allowed for hands-on practice with chest compressions, airway positioning maneuvers, and bag-mask ventilation. The manikins provided tactile feedback on adequate compressions via a “click” indicating appropriate depth. An additional pediatric airway simulation manikin was utilized to teach hands-on endotracheal tube intubation and laryngeal mask placement, and additional airway positioning skills. Skill stations were utilized for participants to practice individual skills. Informal assessment of skills (compressions, bag-mask ventilation, as well as more advanced airway skills) was done in real time with one-on-one teaching with students at skills stations.

To facilitate the sustainability of this intervention, administrative meetings with hospital leadership were held to identify the best method for activating and training an organized hospital-wide resuscitation team. The collective decision was made to implement an on-call rapid response team consisting of a physician and nurse. Hospital staff were instructed to call for a rapid response team for any suspected or impending arrest cases. To build a team of qualified responders, super-users were identified and received further training on resuscitation and communication skills and tasked with future implementation of the curriculum through the continuing medical education program at JFD. Plans were made to continue education every other month. The manikins remained at the hospital after the training team left to allow the super users to continue training more staff.

### 2.3. Assessment Survey

Identical quality assurance surveys were given to the participants before and after the curriculum was taught. Participants who provided written consent were included in the study. Questions assessing provider comfort level and knowledge were included. A Likert scale with ratings 1 through 5 was utilized for the questions assessing provider comfort. Though validated assessment questions for NRP are available, the knowledge assessment component was written by the course developers to highlight the most relevant topics to the staff at JFD, and to keep the survey short enough to be completed in the allotted time. The surveys were also utilized post-intervention as a teaching tool to draw attention to key topics.

Plans were initially made to evaluate knowledge retention by conducting the assessment again six-months and one-year post-intervention, but unfortunately, the Ebola epidemic rendered this plan impractical.

### 2.4. Statistical Analysis

Data was collected on hardcopy surveys and converted to a digital format on Microsoft Excel 16.16.8 spreadsheets (Redmond, WA, USA). Statistical analysis was performed using STATA version 12.0 (College Station, TX, USA). Likert scale results pre- and post-intervention were compared with Wilcoxon signed-rank tests, and knowledge question results were compared using proportions tests and paired *t*-tests. A logistical regression model was utilized to account for potential demographic confounders in comparing pre- and post-intervention test scores.

### 2.5. Ethical Review

The Johns Hopkins School of Medicine Institutional Review Board provided ethical review and approved this study (#IRB00031664).

## 3. Results

There were 75 course participants; 56 individuals completed both the pre-intervention and post-course surveys (Appendix A). Students included technicians, nurses, and physicians (Table 1). Of the 21 participants who regularly took care of neonates, over half (57%) did not have prior cardiopulmonary resuscitation (CPR) training, and only 29% received this training in the past 2 years.

The aggregated median pre-intervention test score was 25% and the median post-intervention test score was 88% (Table 2). On average, participants improved 63% in knowledge assessment scores (*p* < 0.00001). Evaluation of post-intervention scores with regression analysis concluded that pre-intervention test score, prior CPR training, age, and occupation did not have a significant effect. Sex was the only significant factor in determining post-intervention score: females comprised 60% of the participants and were more likely to score higher. However, sex was not affected by occupation, age, or prior CPR training. High pre-intervention test scores, age, sex, occupation, and prior CPR training did not have an effect on the post-intervention test score.

The median provider comfort level score was four pre-intervention, and five post-intervention. An ordered logistic regression analysis concluded that prior CPR training, age, sex, occupation, and post-intervention test scores did not have a significant effect on provider comfort level with neonatal resuscitation after the intervention. A Wilcoxon signed-rank test showed an expected difference in provider comfort level before and after the curriculum (*p* < 0.00001).

## 4. Discussion

As Liberia makes continued efforts to meet its SDG target of decreasing neonatal mortality, research to inform contextually appropriate, effective interventions will be crucial. Though various standard resuscitation protocols exist (i.e., PALS, ACLS, NRP, HBB), the actual training of healthcare providers in resource-limited settings varies by necessity based on the baseline knowledge and variety of practice providers, practice context, availability of subspecialists, equipment, proximity to higher level centers, and even the limited time of providers often working in overburdened and understaffed environments.

Standard protocols often need some adjustment, even if simply in the modification of the instructor strategy or lecture highlights to fit the learners’ time constraints. Additionally, staff attitudes toward the implementation of protocols, communication, and teamwork are key to emphasize during training to have local acceptability [13]. For example, neither only HBB or only NRP would have been the right combination for JFD’s context, which is a hospital with enough resources and personnel to be able to provide NRP-level of care, but with the majority of newborns being born in the community that would benefit from being cared for by attendants with HBB training. It is also notable that although most participants had not been previously trained in neonatal resuscitation and scored lower on the pre-intervention test, their initial comfort levels with their neonatal resuscitation skills were fairly high, indicating that the need for training may be higher than perceived. This study suggests that even within a modified resuscitation curriculum, neonatal resuscitation can be effectively taught and practiced with a demonstrated improvement in knowledge and comfort level. In addition, there is a paucity of literature on Liberia-specific neonatal resuscitation training, and this study provides some preliminary evidence. Studies conducted in other parts of Africa with similar resource constraints also show positive results in knowledge improvement [14,15,16].

This study found that occupation, age, or prior CPR experience did not have a significant effect on pre- or post-intervention test scores. This suggests that our curriculum had similar effectiveness across various demographics, and that holding the same training for various provider types may be effective, even in a hospital-wide setting. This is particularly significant for the resource-limited setting, as often providers find themselves functioning by necessity in multiple roles with varying amounts of specialized training. However, this study’s sample size limits the generalizability of this conclusion, and future work should emphasize addressing potential learning differences in the providers receiving the training.

While this study provides initial data, our next steps will be to further assess objective outcomes. Next steps should include assessing neonatal mortality rates in the catchment region, validated assessments of provider skill abilities (both in situ and in simulation), and tracking provider skill and knowledge retention over time. Research in learning theory suggests that a combination of testing and spaced repetition may be the most effective for learning retention and may enhance the learner’s ability to generalize their knowledge to new situations [17]. While the optimal spacing has not been established in the literature for neonatal resuscitation, Cavicchiolo et al. [16] in Mozambique suggest low-dose/high-frequency training with an adapted NRP curriculum may improve initiation and time to resuscitation [16]. Further work should focus on establishing an evidence base for the optimum spacing and delivery method for neonatal resuscitation training, as sustainability will be crucial to enacting a lasting effect on the overall mortality rate in Liberia.

The most significant limitation of this study is that these results are not easily generalizable, given the modified curriculum, limited validity questionnaire, and small sample size. However, this still makes a relevant point that even when taught a modified neonatal resuscitation curriculum, resuscitation knowledge and proficiency with neonatal resuscitation skills can be learned. This is especially poignant in resource-limited settings, as often pediatricians or neonatologists may not be readily available, and every practitioner may need to maintain a broader skill set in resuscitation than those practicing in highly specialized and resourced contexts.

## 5. Conclusions

A modified NRP and manikin simulation-based curriculum may be an effective way of teaching health care providers in resource-limited settings. Training of providers in resource-limited settings could potentially help decrease neonatal mortality in Liberia. Modification of protocols is sometimes necessary and an important part of providing context-specific training. The results of this study have no direct relation to decreasing neonatal mortality until proven. A general resuscitation curriculum with modified NRP training may be effective, and further work should focus on the effect of such interventions on neonatal mortality rates in the region.

## Figures and Tables

**Table 1:** Demographics of course participants.

Demographic	Number of Participants (*n* = 56)
**Age (years)**	Mean 36.2
20–29	10 (17.9%)
30–39	31 (55.4%)
40–49	8 (14.3%)
50–59	4 (7.1%)
60–69	1 (1.8%)
Missing	2 (3.6%)
**Male Sex**	22 (39.3%)
**Occupation**	
Physician	12 (21.4%)
Nurse	31 (55.3%)
Technician	6 (10.7%)
Midwife	3 (5.4%)
Physician assistant	2 (3.6%)
Pharmacist	1 (1.8%)
Nurse Anesthetist	1 (1.8%)
**Prior Neonatal Resuscitation Experience**	
Yes	21 (37.5%)
No	35 (62.5%)
**Regular Neonatal Care**	
Yes	21 (37.5%)
No	35 (62.5%)

**Table 2:** Pre- and post-intervention assessment scores.

Median Knowledge Assessment Scores	
Pre-intervention	25% (25–38)
Post-intervention	88% (63–88)
Improvement	63% (*p* < 0.00001)
**Provider Comfort Level Scores**	**Scale 1–5**
Pre-intervention	4 (SD = 1.4)
Post-intervention	5 (SD = 0.4)

SD: standard deviation.

## Appendix A. Pre- and Post-Intervention Assessment

1. I am confident that I can teach someone to recognize when a neonate may need CPR (1–5 Likert scale).
2. How comfortable are you with performing CPR on a neonate (1–5 Likert scale)?
3. Which of the following should be done within 30 s of delivery?
  (a) Start chest compressions.
  (b) Ventilate.
  (c) Open the airway.
  (d) Calculate APGAR score.
4. At 30 s post-delivery, if a neonate has a heart rate < 10 and is apneic, how quickly should you assist him/her with positive pressure ventilation?
  (a) 100 breaths/min.
  (b) 40–60 breaths/min.
  (c) 10–20 breaths/min.
  (d) 60–80 breaths/min.
5. If peak inspiratory pressures > 20 are needed for positive pressure ventilation, what are you likely to need?
  (a) Pulse oximetry
  (b) Chest tube
  (c) Endotracheal tube
  (d) Orogastric tube.
6. What FiO_2_ should you initially use if positive pressure ventilation is needed in a full-term baby?
7. After 60 s of life, if the baby’s HR is <100 and you have provided adequate positive pressure ventilation, how many compressions per minute should be provided?
8. After how many chest compressions should you give 1 breath?
9. If needed, what dose of epinephrine should you give intravenously initially?
10. A neonate has a heart rate of 60, slow respirations, is limp but grimacing, and has blue extremities but is otherwise pink. Calculate his APGAR score.
